# Antioxidant Effects and Potential Mechanisms of *Citrus reticulata* ‘Chachi’ Components: An Integrated Approach of Network Pharmacology and Metabolomics

**DOI:** 10.3390/foods13244018

**Published:** 2024-12-12

**Authors:** Jiahao Xiao, Tian Sun, Shengyu Jiang, Zhiqiang Xiao, Yang Shan, Tao Li, Zhaoping Pan, Qili Li, Fuhua Fu

**Affiliations:** 1 Hunan Agriculture Product Processing Institute, Hunan Academy of Agricultural Sciences, Changsha 410125, China; jiahaoxiao@foxmail.com (J.X.);; 2 Zheng Gan Hui (Jiang Men Xin Hui) Dried Tangerine Peel, Ltd., Jiangmen 529100, China; 3 Longping Branch, College of Biology, Hunan University, Changsha 410125, China

**Keywords:** *Citrus reticulata* ‘Chachi’, metabolomics, flavonoids, network pharmacology, antioxidant activity

## Abstract

*Citrus reticulata* ‘Chachi’ (CRC), recognized for its considerable edible and medicinal significance, is a valuable source of metabolites beneficial to human health. This research investigates the metabolic distinctions and antioxidant properties across four different parts of CRC, using multivariate statistical analysis to interpret metabolomic data and network pharmacology to identify potential antioxidant targets and relevant signaling pathways. The results indicate considerable metabolic differences in different parts of the sample, with 1622 metabolites showing differential expression, including 816 secondary metabolites, primarily consisting of terpenoids (31.02%) and flavonoids (25.22%). The dried mature citrus peel (CP) section demonstrates the highest level of total phenolics (6.8 mg/g), followed by the pulp without seed (PU) (4.52 mg/g), pulp with seed (PWS) (4.26 mg/g), and the seed (SE) (2.16 mg/g). Interestingly, targeted high-performance liquid chromatography of flavonoids reveals the highest level of nobiletin and tangeretin in CP, whereas PU has the highest level of hesperidin, narirutin, and didymin. Furthermore, all four sections of CRC exhibit robust antioxidant properties in in vitro assessments (CP > PU > PWS > SE). Lastly, the network pharmacology uncovered potential antioxidant mechanisms in CRC. This research offers deeper insights into the development and utilization of byproducts in the CRC processing industry.

## 1. Introduction 

*Citrus reticulata* ‘Chachi’ is mainly planted and harvested in Xinhui, Guangdong, China [1]. Various parts of the fruit (peel, pulp, and seeds) are valuable sources of a variety of bioactive compounds, and the dried mature peel is known as “Guang Chen Pi” (CP) [2]. CP is abundant in various bioactive substances, including phenolic acids, terpenoids, and flavonoids, and has been extensively documented in the Chinese Pharmacopoeia for treating diseases like cough and indigestion [3,4]. Additionally, it is widely used as a medicinal food and in fragrances, cosmetics, and other fields [5]. Although the pulp and seeds of CRC are often discarded as processing byproducts, they are rich in phenolic and terpenoid bioactive compounds. However, the exploration of the nutrient composition and functional activity of the pulp (including seeds) of CRC is still far from sufficient. Further, plant-based chemical substances from natural sources exhibiting bioactivity are crucial for promoting human health through the prevention and treatment of diseases [6]. These compounds exhibit potential antioxidant properties, which can reduce the probability of chronic disease by inhibiting the reactive oxygen species (ROS) [7]. Therefore, the utilization of the peel and the transformation of its byproducts into high-nutrition dietary supplements or functional foods are effective ways to achieve high-value applications of citrus resources. To maximize the use of CRC, research has focused on the chemical components and bioactivity of different parts of CRC. Although many studies have concentrated on the isolation and characterization of flavonoids from the peel [8,9,10,11], the differential analysis and comparison of pulp with CP has rarely been studied, so whole-fruit utilization of CRC has not been realized. To promote the high-value utilization of CRC pulp (including seeds) and increase its potential economic value, understanding its main differentially abundant metabolites and functional activities and comparing its significant differences in composition and efficacy with those of CP are crucial.

Targeted and non-targeted metabolomic approaches based on liquid chromatography–tandem mass spectrometry (LC-MS-MS) measurements have become vital research tools in food science [6]. Non-targeted metabolomics can identify many small-molecule metabolites in samples, revealing compositional differences by comparing metabolite variations. Moreover, targeted metabolomics focuses on the quantitative analysis of specific secondary metabolites in samples, further validating the results obtained from non-targeted metabolomic analyses [12,13,14].

With recent advances in bioinformatics, network pharmacology has been increasingly used in scientific research, particularly in the exploration of traditional Chinese medicinal herbs with multi-component and multi-target characteristics [12]. Network pharmacology describes complex links among drug components, disease targets, and pathways. For example, Peng et al. identified eight key antioxidant compounds and five core antioxidant targets by integrating metabolomics and network pharmacology to uncover the main antioxidants and their underlying mechanisms in Chinese Cabernet Sauvignon red wine; Yu et al. reported key metabolites and targets for the antibacterial effects of *Castanopsis* honey; and Li et al. reported crucial compounds for the treatment of ulcerative colitis in the peel of *Citrus reticulata* ‘Dahongpao’ via an integrated approach [15,16,17] (Li et al., 2024; Peng et al., 2024; Yu et al., 2024).

In this study, metabolomic analysis revealed the basis of the differences in the bioactivity of chemical components among various parts of *Citrus reticulata* ‘Chachi’, and the antioxidant activities were compared. Further, by integrating metabolomic and network pharmacology analyses, the antioxidant mechanisms of flavonoids in CRC were predicted. These findings enhance the understanding of the active components and health benefits of the mature dried peel, pulp, and seeds of CRC. Additionally, it establishes a theoretical foundation for the complete use of chenpi, pulp, and seeds.

## 2. Materials and Methods

### 2.1. Chemical Reagents

Methanol, acetonitrile, formic acid, and propanol (HPLC grade, ≥99.9%) were purchased from KeMeiOu Company (Tianjin, China), and water was purchased from China Resources Yibao (Shenzhen, China). Analytical standards of verbascoside, taxifolin, naringin, hesperidin, neohesperidin, rutin, luteolin, diosmin, artemisin, acacetin, chrysin, osmanthus flavone, chrysoeriol, tangeretin, and gallic acid (all HPLC grade, ≥98.0%) were obtained from DeSt Biotech Co., Ltd. (Chengdu, China). Folin–Ciocalteu reagent was obtained from BoMei Biotech Co., Ltd. (Hefei, China). All the chemicals and solvents were purchased from Sinopharm (Beijing, China).

### 2.2. Sample Preparation

As shown in Table 1, the CRC’s pulp and dried peel samples used in the experiment were procured from Jiangmen City, Guangdong Province. The dried peel samples had been naturally aged for three years. The pulp of *Citrus reticulata* was harvested in December 2023, with sample information detailed in Table 1. Initially, the pulp was kept in a −40 °C freezer for 5 h for pre-freezing, followed by the entire fruit being placed into a vacuum freeze dryer (FD-205m2, Dongming, Zhangzhou, China) for drying treatment for 24 h at a vacuum pressure of 80 Pa. Subsequently, the obtained dried citrus pulp was divided into two parts: one part without the seeds, while the other part was kept with seeds intact. After that, all the samples, including CP, PWS, PU, and SE, were ground into fine powder using a high-speed grinder (JB-30B, Baoli, Jiangyin, China). Subsequently, the samples were sifted through a 60-mesh sieve and stored at 4 °C until further use.

### 2.3. Untargeted Metabolomic Analysis

#### 2.3.1. Metabolite Extraction

An amount of 50 mg of the sample was taken into a centrifuge tube containing grinding balls. Next, 400 µL of the prepared methanol extraction solution, which has a ratio of 4:1 by volume of methanol to water and contains 0.02 mg/mL of L-2-chlorophenylalanine, was used as an internal standard. The sample was ground for 10 min at −10 °C and 50 Hz using the Wonbio-96c type cryogenic tissue grinder (Wonbio Biotechnology, Shanghai, China). Then, ultrasound extraction was performed for 20 min at 4 °C and 35 kHz in the SBL-12TD ultrasonic cleaner (model 300W-10L, Xinzhi Biotechnology, Ningbo, China). The sample was kept undisturbed at −24 °C for 35 min. Subsequently, the sample was centrifuged at 4 °C and 13,000 rpm for 10 min (Centrifuge 5430R, Eppendorf, Hamburg, Germany). The supernatant was transferred into the injection vial for online analysis [18].

#### 2.3.2. Untargeted Metabolomic Analysis via Ultra-High-Performance Liquid Chromatography (UHPLC)–Tandem Mass Spectrometry (MS/MS)

The chromatographic separation was performed on a HSS T3 column (1.8 μm, 2.1 mm × 100 mm, Waters, Milford, MA, USA) with the following conditions: injection volume: 2 μL; column temperature: 40 °C; solvent A: water (containing 0.01% formic acid); solvent B: 45% acetonitrile, 45% isopropanol, and 10% water; gradient elution procedure: 0–2 min, 0–10% of mobile phase B; 2–3.5 min, 10–35% of mobile phase B; 3.5–5 min, 35–65% of mobile phase B; 5–7 min, 65–100% of mobile phase B; 7–8 min, maintain 100% of mobile phase B; 8–8.5 min, 100–50% of mobile phase B; 8.5–9 min, 50–0% of mobile phase B; 9–10 min, maintain 0% of mobile phase B; flow rate: 0.4 mL/min; detector: mass spectrometry in both positive and negative modes at voltages of 3500 V and 4500 V, respectively, with a *m*/*z* range of 70–1050 Da; auxiliary gas heater temperature: 300 °C; ion transfer tube temperature: 320 °C; sheath gas: 50 psi; auxiliary gas at: 10 psi; collision energy cycle range: 20–40–60 V; resolution for MS1 and MS2: 60,000 and 10,000, respectively [19,20].

### 2.4. In Vitro Antioxidant Capacity

#### 2.4.1. Extraction of Phenolic Compounds

Weigh 1 g of the sample and place it in a triangular flask, add 3 mL of 70% methanol solution, extract under ultrasonication in the dark for 30 min (25 °C, 40 kHz), and then transfer to a 10 mL centrifuge tube and centrifuge for 10 min (4 °C, 12,000 rpm) (Centrifuge 5430R, Eppendorf, Hamburg, Germany). The supernatant was collected, and the residue was re-extracted twice using the same protocol. Finally, the total volume was adjusted to 10 mL for phenolic content and antioxidant activity assays [20].

#### 2.4.2. Analysis of Total Phenolic Content (TPC)

The Folin–Ciocalteu assay was used to determine TPC in the samples. Briefly, 0.2 mL of the aforementioned extract was transferred into a 15 mL centrifuge tube, to which 2.3 mL of Folin–Ciocalteu reagent and 0.5 mL of 7.5% Na_2_CO_3_ solution were added. After thorough mixing, the samples were kept in the dark for two hours. The absorbance was recorded at 760 nm with a microplate reader (SYNERGY H1, BioTek, Winooski, VT, USA). TPC was quantified using standard curve for gallic acid [21]. The range of concentrations for the standard curve was 0 to 80 μg/mL.

#### 2.4.3. Targeted Determination of Flavonoids

Flavonoid levels were ascertained via high-performance liquid chromatography (HPLC) using the chromatographic conditions as described previously with some modifications [22]. HPLC conditions used as follows: ZORBAX SB-C18 analytical column (4.6 mm × 250 mm, 5 μm, Agilent, Santa Clara, CA, USA); mobile phase (A: water with 0.1% (*v*/*v*) formic acid and B: methanol); elution conditions: 0–20 min, 37–50% B; 21–35 min, 50–80% B; 36–40 min, 80–100% B; 41–50 min, 100–37% B; column temperature: 25 °C; injection volume: 3.0 μL; total flow rate: 0.3 mL/min; quantification wavelengths: 283 nm (for verbascoside, taxifolin, naringin, hesperidin, neohesperidin, rutin, luteolin, diosmin, artemisin, acacetin, chrysin, osmanthus flavone, chrysoeriol, tangeretin, and gallic acid) and 330 nm (for diosmetin, sweet orange flavone, chenpi flavone, tangerine flavone, and chrysoeriol). The calibration curves for mixed standards were generated using the external standard technique, which relied on retention time and peak area measurements [12]. The detection limit (LOD) and the quantitation limit (LOQ) were calculated using the formulas specified in the ICH guideline Q2B.

#### 2.4.4. 2,2-Diphenyl-1-picrylhydrazyl (DPPH) Radical Scavenging Activity Assay

The antioxidant activity of the extract was measured using a DPPH detection kit (Komen Biotech, Suzhou, China) by following the standard protocol, and absorbance measurements were taken at 515 nm with a microplate reader (Synergy H1, BioTek, VT, USA).

#### 2.4.5. 2,2′-Azinobis-(3-ethylbenzothiazoline-6-sulfonic Acid (ABTS) Radical Scavenging Activity Assay

The antioxidant activity of the extract was assessed using an ABTS detection kit (Komen Biotech, Suzhou, China) by following the standard protocol, and the absorbance measurements were carried out at 734 nm with a microplate reader.

#### 2.4.6. Ferric Reducing Antioxidant Power (FRAP) Assay

The antioxidant nature of the extract was evaluated using a FRAP detection kit (Komen Biotech, Suzhou, China), and the absorbance was measured at 760 nm with a microplate reader.

### 2.5. Network Pharmacology

#### 2.5.1. Prediction of Flavonoid Active Components and Core Targets

The SwissADME database (http://www.swissadme.ch/, accessed on 1 August 2024) was utilized to predict the pharmacokinetics characteristics of the active components from the flavonoids identified through targeted HPLC detection. Components with a drug-likeness (DL) score ≥0.14 are considered potential active constituents of *Citrus reticulata* ‘Chachi’ [20]. Subsequently, the SwissTargetPrediction database (http://www.swisstargetprediction.ch/, accessed on 1 August 2024) was employed to obtain the most probable protein targets for these active components. The 2D structures of the active components in the MOL format were retrieved from the LIPID MAPS^®^ database (https://www.lipidmaps.org/, accessed on 2 August 2024) and submitted to SwissTargetPrediction. The species selected was “*Homo sapiens*”, and targets with a probability greater than 0 were considered potential targets.

#### 2.5.2. Prediction of Core Targets for Oxidative Damage

Disease-related gene targets associated with oxidative damage were retrieved from the OMIM (https://www.omim.org/, accessed on 5 August 2024) and GeneCards (https://www.genecards.org/, accessed on 5 August 2024) databases using the keyword “oxidative damage”. Subsequently, duplicates were removed, and the data from both databases were merged for further use. A further intersection analysis of the oxidative stress-related and flavonoid-active targets via a Venn diagram was carried out to identify the shared targets [23].

#### 2.5.3. Construction of Protein–Protein Interaction Network and Key Gene Selection

STRING (https://www.string-db.org/, accessed on 5 August 2024) database was employed to analyze the protein–protein interaction networks. The overlapping targets obtained from the Venn diagram analysis were imported into the STRING database with “*Homo sapiens*” selected as the species, and then the TSV file was downloaded. In Cytoscape 3.7.1, the TSV file was imported and the MCC analysis algorithm in Cytohubba was used to identify the top 10 hub genes [23].

#### 2.5.4. GO and KEGG Enrichment Analysis

For a deeper examination of various biological processes, cellular components, molecular functions, and signaling pathways related to oxidative damage, the overlapping targets were submitted to the David database (https://david.ncifcrf.gov/, accessed on 6 August 2024) for GO and KEGG enrichment analyses (Table S1). The identification and species selected were “OFFICIAL_GENE_SYMBOL” and “Homo sapiens”, respectively, with *p* < 0.05 considered as statistically significant.

### 2.6. Data Analysis

The untargeted metabolomics data were replicated six times, with other datasets in triplicate, and presented as mean ± SD. Statistical analyses were conducted using SPSS Statistics (version 26.0) by employing one-way ANOVA and Duncan’s test for group comparisons. Multivariate statistical methods were used for metabolomics data analysis, and a *p*-value < 0.05 was considered statistically significant. Further, PCA analysis, partial least squares discriminant analysis (PLS-DA), and permutation tests were performed in SIMCA (14.1, Umetrics Umea, Sweden). For visualization, heat maps were plotted using the Pheat map package (v.1.0.8) in R.

## 3. Results and Analysis

### 3.1. Identification of Metabolites

**Figure 1:**
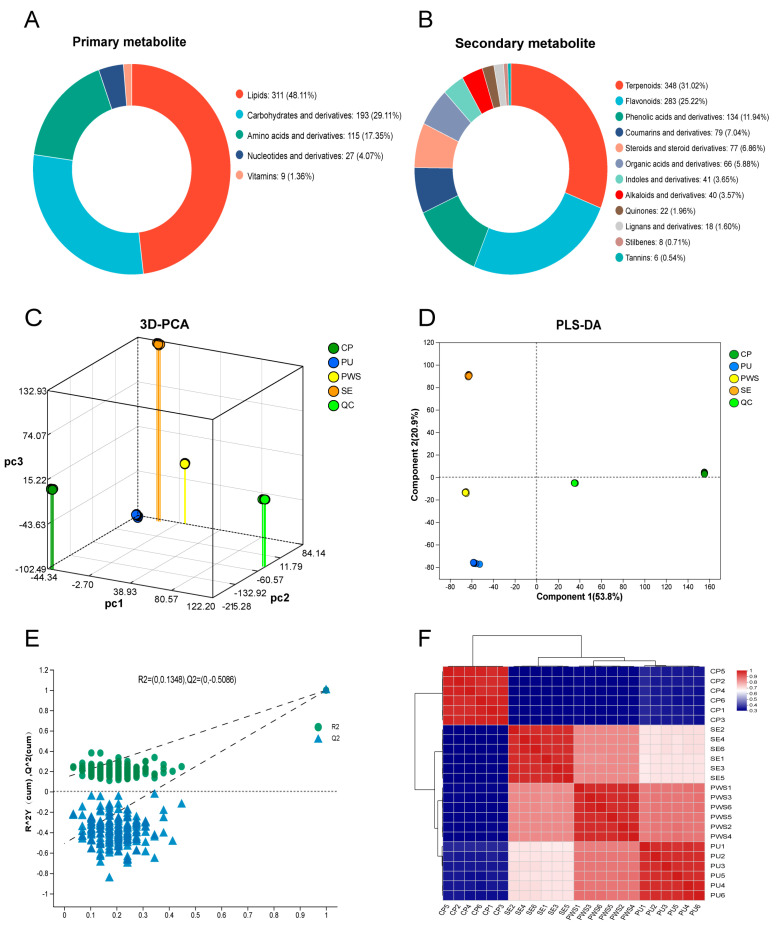
(**A**) Classification of primary metabolites. (**B**) Classification of secondary metabolites. (**C**) Three-dimensional PCA score plot. (**D**) PLS-DA score plot. (**E**) Permutation test plot with 200 permutations. (**F**) Sample correlation heat map.

As shown in Figure 1, numerous compounds were identified in positive and negative ionization modes through untargeted metabolomics, detecting 13,845 and 12,180 mass spectrometry peaks, respectively. A total of 2329 metabolites were detected in positive and negative ion modes, including lipids (319), carbohydrates and derivatives (193), amino acids and derivatives (115), terpenoids (348), flavonoids (283), phenolic acids and derivatives (134), coumarins and derivatives (79), steroids and derivatives (77), and additional metabolites (781) (Figure S1 and Figure 1A,B, Table S2). Additionally, more peaks were detected in the positive ion mode compared to the negative ion mode, corroborating the literature [12]. Prior research has shown that phenolic acids, flavonoid compounds, and fatty acids produce stronger signals in the negative ion mode, while anthocyanins, coumarins, limonoids, and nitrogen-containing compounds exhibit better ionization in the positive ion mode [20].

### 3.2. Metabolomic Analysis

A quality control (QC) sample was prepared by pooling aliquots from all samples for quality control analysis. A total of twenty-four samples were subjected to metabolomic analysis, including six biological replicates for each of the four tissues of *Citrus reticulata* ‘Chachi’ [pulp without seed (PU), pulp with seed (PWS), dried mature citrus peel (CP), and seed (SE)].

PCA analysis assessed the metabolic differences among and within the groups of *Citrus reticulata* ‘Chachi’. The results revealed that the first principal component accounted for 50.60% and the second for 23.20% of the variance (Figure 1C, Table S3). Distinct group separation was observed, which highlighted metabolic disparities across the CRC’s parts. The tight clustering of quality control samples in the PCA analysis assured the consistency and accuracy of the analytical platform, with reliable sample replication across the four groups.

PLS-DA, a supervised discriminant analysis method, can eliminate systematic errors caused by the environment and other factors, resulting in better separation effects than PCA [13]. The PLS-DA score plot (Figure 1D) showed complete separation among the four groups of samples and QC sample points without overlapping areas, which indicates apparent differences between different parts of CRC. Similar to PCA, PLS-DA can significantly distinguish CRC samples from other tissues. Further, a random permutation test (200 times) was performed to prove the accuracy and validation of the PLS-DA model. The permutation test plot (Figure 1E) revealed R2 and Q2 values of 0.1348 and −0.5086, respectively, suggesting no overfitting and demonstrating a stable and reliable model. The sample correlation heat map (Figure 1F) showed that the correlation coefficients were arranged in descending as PU > PWS > SE > CP, and correlations approaching 1 suggest more significant metabolic similarity between samples. Therefore, PU has the highest correlation, followed by PWS and SE, while CP demonstrated the lowest correlation.

### 3.3. Selection and Identification of Differential Metabolites

**Figure 2:**
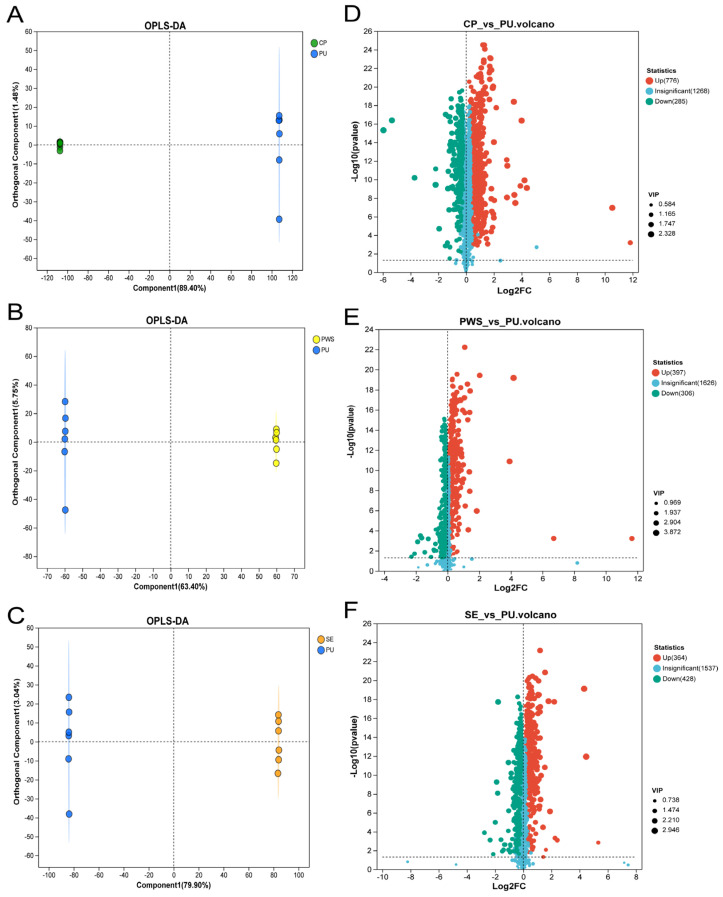
(**A**–**C**) OPLS-DA score plots. (**A**) CP and PU; (**B**) PWS and PU; (**C**) SE and PU. (**D**–**F**) Volcano plots of differential metabolite expression levels. (**D**) CP and PU; (**E**) PWS and PU; (**F**) SE and PU.

Orthogonal partial least squares discriminant analysis (OPLS-DA) was applied to identify differential metabolites in the pairwise comparisons of CP, PWS, SE, and PU, as depicted in Figure 2. The obtained Q2 and R2Y values surpassed 0.9 (Figure S2), signifying a robust model [24]. The OPLS-DA score plots (Figure 2A–C) clearly showed distinctions among citrus tissues, suggesting variations in their metabolomic profiles. Further, differential metabolites were selected based on a VIP threshold (>1) and a *p*-value (<0.5) and were visualized using volcano plots [25]. In comparison to PU, the analysis revealed 1061 (776 upregulated and 285 downregulated), 703 (397 upregulated and 306 downregulated), and 792 (364 upregulated and 428 downregulated) differential metabolites in CP, PWS, and SE, respectively.

**Figure 3:**
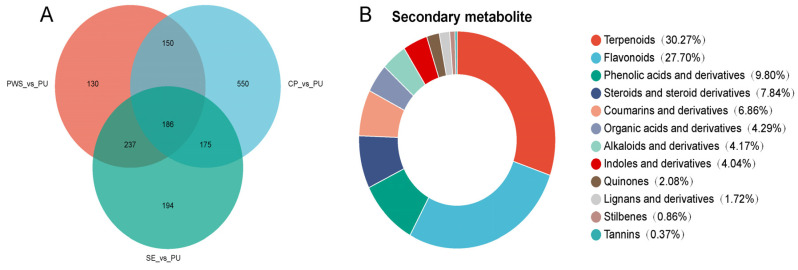
(**A**) Venn diagram of differential metabolites; (**B**) Classification of 816 secondary differential metabolites.

Venn diagrams (Figure 3A) illustrate the relationships between differentially expressed metabolites across the various groups (PWS vs. PU, CP vs. PU, and SE vs. PU). These diagrams were constructed using the VIP values from OPLS-DA and the *p* values from t-tests to screen for differentially expressed metabolites among the groups. A total number of 1622 differential metabolites were identified across the Venn diagrams, with PWS vs. PU, CP vs. PU, and SE vs. PU groups exhibiting 703, 1061, and 792 differential metabolites, respectively. From the 1622 differential metabolites, 816 secondary metabolites were identified, which were categorized as terpenoids (247, 30.27%), flavonoids (226, 27.7%), phenolic acids (80, 9.80%), steroids and their derivatives (64, 7.84%), coumarins and their derivatives (56, 6.86%), organic acids (35, 4.29%), alkaloids and their derivatives (34, 4.17%), indole and its derivatives (33, 4.04%), quinones (17, 2.08%), lignans and their derivatives (14, 1.72%), quinone and its derivatives (7, 0.86%), and tannins (3, 0.37%) (Figure 3B). Among these, terpenoids and flavonoids were identified as the predominant secondary metabolites with various biological activities, including antioxidant and anti-inflammatory effects [8,26,27,28,29]. Furthermore, phenolic acids and coumarins were recognized for their potential biological activities, such as antimicrobial and antioxidant activities [26,30,31].

### 3.4. Comparative Analysis of Secondary Differential Metabolites

**Figure 4:**
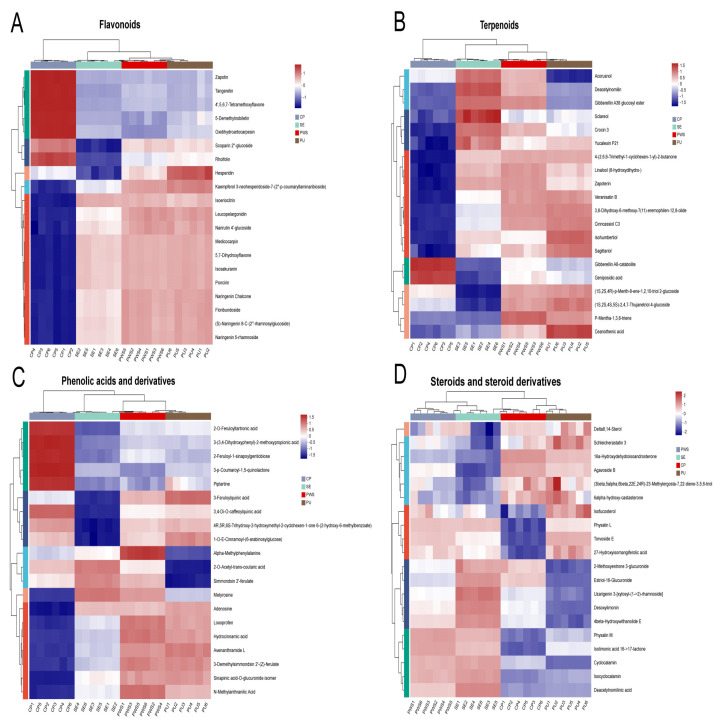
Heat maps display the levels of secondary differentially expressed metabolites in four parts of CRC. (**A**) Flavonoids; (**B**) Terpenoids; (**C**) Phenolic acids and derivatives; (**D**) Steroids and steroid derivatives.

Further, we generated eight heat maps for the secondary differential metabolites by selecting the top 20 differentially expressed metabolites for clustering, which allowed us to visualize the trends in differential metabolites across different groups (Figure 4A–D. The heat map displays samples in columns and metabolites in rows, where the red color indicates upregulated, and the blue color presents downregulated metabolites.

#### 3.4.1. Flavonoids

Flavonoids are the predominant phenolic constituents found in citrus. They significantly contribute to the management of chronic disease by reducing oxidative stress and inflammation [32]. Our analysis identified 149 (121 upregulated and 28 downregulated), 118 (71 upregulated and 47 downregulated), and 108 (76 upregulated and 32 downregulated) differential flavonoids in the CP, SE, and PWS versus the PU groups, respectively (Tables S4–S6). Further, the heat map showed (Figure 4A) Zapotin, Tangeretin, 4′,5,6,7-Tetramethoxyflavone, 5-Demethylnobiletin, Scoparin 2″-glucoside, and Rhoifolin in the CP group and Oxidihydroartocarpesin had the highest relative content. Most of these flavonoids were polymethoxyflavonoids (PMFs) found only in citrus and are polyphenols with strong antioxidant properties [33]. Several studies have reported various potential pharmacological activities of PMFs; for example, they can inhibit inflammation in different in vitro and in vivo models [34]. In addition, the results showed that flavonoid compounds such as Hesperetin, Isoeriocitrin, 4′-glucoside, Leucopelargonidin, Narirutin, Medicocarpin, and Poncirin had the highest relative content in the PU group and most of them belonged to flavanones. Finally, the CP group exhibited a significantly higher relative PMF expression while the SE group showed the lowest among flavonoids.

#### 3.4.2. Terpenoids

Research has shown that terpenoids have strong antioxidant properties that scavenge free radicals and protect cells from oxidative damage [35,36]. In addition, some studies have reported antiproliferative activity of triterpenoids against cancer cells [37]. Terpenoids have received much attention from researchers for their pharmacotherapeutic activity, such as antioxidant and antitumor properties. In this study, we found that Acorusnol, Deacetylnomilin, Gibberellin A38 glucosyl ester, Sclareol, and Crocin 3 had the highest relative content in the SE group (Figure 4B), followed by the PWS group. Overall, the CP group exhibited a lower abundance of differential terpenoids than the SE, PWS, and PU groups.

#### 3.4.3. Phenolic Acids and Derivatives

Phenolic acids constitute a significant class of phenolics naturally present in citrus, and many of them have been reported to exhibit potent antioxidant properties [38]. The findings of the analysis revealed 55 (42 upregulated, 13 downregulated), 35 (10 upregulated, 25 downregulated), and 20 (16 upregulated, 4 downregulated) differential phenolic acids in the CP, SE, and PWS groups versus the PU group, respectively (Tables S7–S9). Phenolic acids, namely 2-O-Feruloyltartronic acid, 2-Feruloyl-1-sinapoylgentiobiose, 3-p-Coumaroyl-1,5-quinolactone, and Piplartine were found to be significantly higher in the CP group in terms of their relative content (Figure 4C), which may be a better source for extracting phenolic acids from the plant.

#### 3.4.4. Steroids and Steroid Derivatives

Steroids are a class of lipid molecules with a tetracyclic structure, and studies demonstrate the anti-inflammatory effects of steroids [39]. The results showed the highest relative content of Isolimonic acid 16->17-lactone, Deacetylnomilinic acid, Cyclocalamin, and 4beta-Hydroxywithanolide E in the SE group, followed by the PWS group (Figure 4D). Moreover, the relative content of 16a-Hydroxydehydroisoandrosterone and Agavoside B were highest in the CP group. Overall, the SE group exhibited more steroid abundance than the PU, CP, and PWS groups.

#### 3.4.5. Coumarins and Their Derivatives

**Figure 5:**
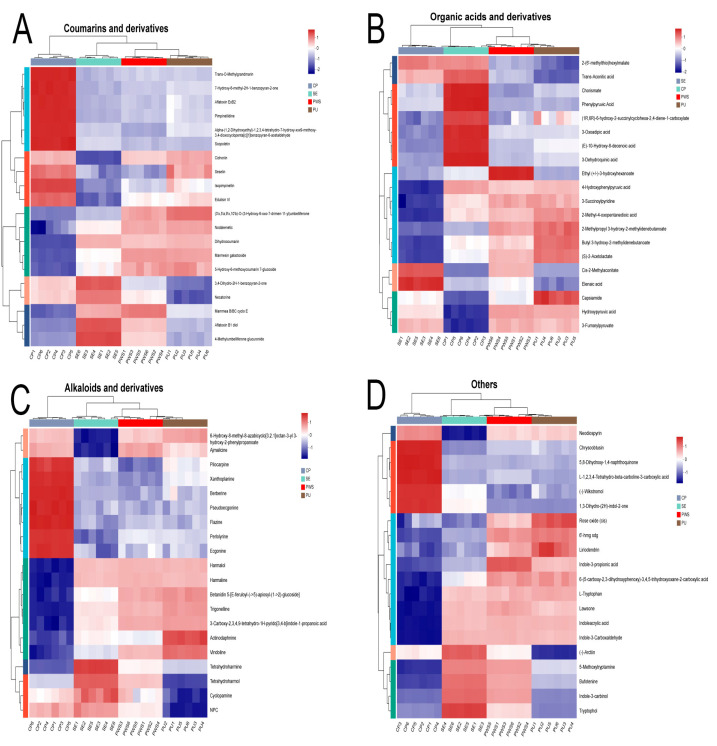
Heat maps display the levels of secondary differentially expressed metabolites in four parts of CRC. (**A**) Coumarins and derivatives; (**B**) Organic acids and derivatives; (**C**) Alkaloids and derivatives; (**D**) Others.

Coumarins attract attention for their diverse therapeutic effects, such as antioxidant and anti-inflammatory actions [40,41]. The results from the analysis detected a relatively high level of coumarins in the CP group, including Trans-O-Methylgrandmarin, Scopoletin, Aflatoxin ExB2, Pimpinellidine, Edulisin VI, and others (Figure 5A). The relative amount of Aflatoxin B1 diol was significantly higher in the SE group than in the other groups, and it may be a potential biomarker.

#### 3.4.6. Organic Acids, Alkaloids and Other Metabolites

Organic acids and alkaloids are active compounds in citrus fruits, which may have potential health benefits for humans [42,43]. The findings showed that organic acids, namely Chorismate, Phenylpyruvic Acid, 3-Dehydroquinic acid, and 3-Oxoadipic acid, were abundant in the CP group, surpassing their levels in other groups. While Cis-2-Methylaconitate and Elenaic acid were most prevalent in the SE group. Also, the relative abundance of Butyl 3-hydroxy-2-methylidenebutanoate, (S)-2-Acetolactate, and 2-Methyl-4-oxopentanedioic acid content was found to be highest in the PU group (Figure 5B). Alkaloids were predominantly upregulated in the CP and PU groups, with twenty upregulated and six downregulated (Table S10). Further, the relative abundance of alkaloids such as Berberine, Pilocarpine, and Xanthoplanine was highest in the CP group (Figure 5C). Additionally, other metabolites including Neodiospyrin, Chrysoobtusin, (-)-Wikstromol, 1,3-Dihydro-(2H)-indol-2-one, and 5,8-Dihydroxy-1,4-naphthoquinone were highly expressed in the CP group (Figure 5D). Rose oxide (cis), 6′-HMG SDG, Liriodendrin, and Indole-3-propionic acid were highly expressed in the PU and PWS groups. 5-Methoxytryptamine, Bufotenine, Indole-3-carbinol, and Tryptopho were highly expressed in the SE and PWS groups with a higher relative content.

### 3.5. KEGG Enrichment Analysis of Secondary Differential Metabolites

**Figure 6:**
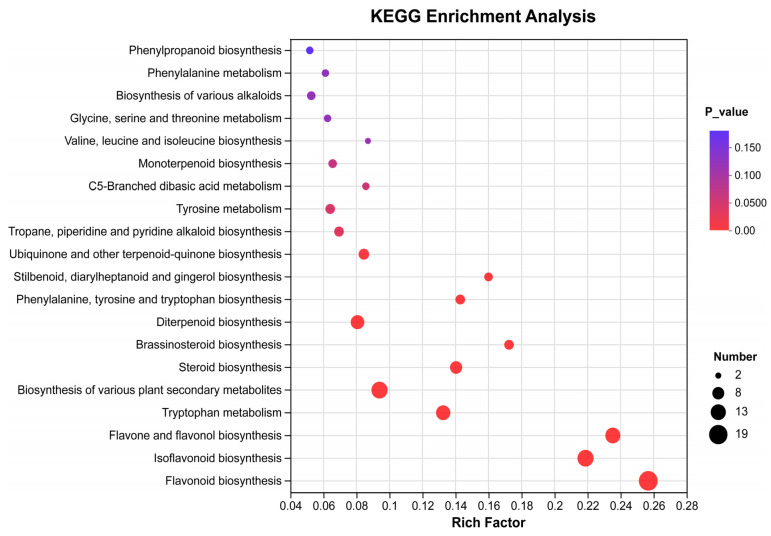
Bubble chart of KEGG enrichment pathways for secondary differential metabolites.

The KEGG database was used to enrich the secondary differential metabolites in the CP, SE, PWS, and PU groups (Figure 6). The secondary differential metabolites in the CP, SE, and PWS versus PU groups were predominantly found across five metabolic pathways: flavonoid biosynthesis, isoflavonoid biosynthesis, flavone and flavonol biosynthesis, tryptophan metabolism, and biosynthesis of various plant secondary metabolites.

### 3.6. Analysis of Flavonoid Compositions in Different Parts of Citrus reticulata ‘Chachi’

**Figure 7:**
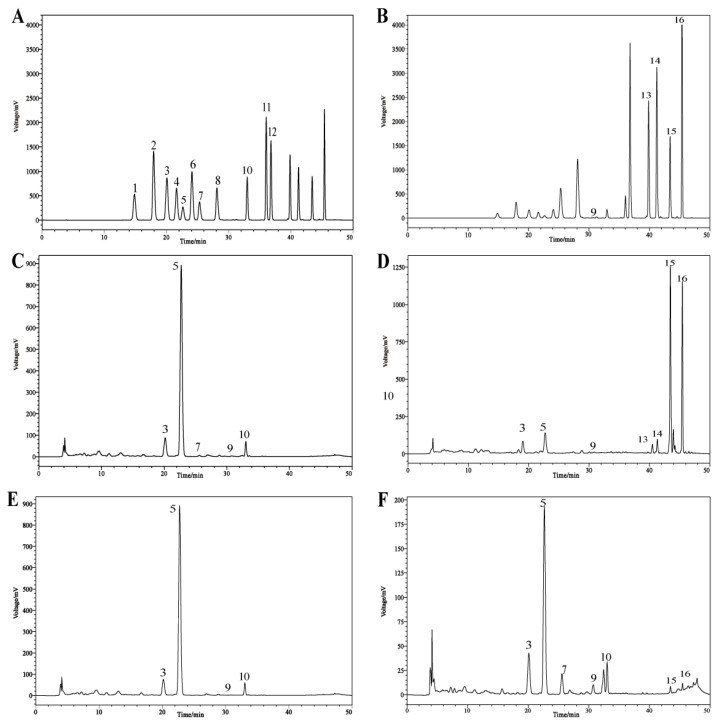
HPLC chromatograms of 16 flavonoid standards (1, Verbascoside; 2, Taxifolin; 3, Narirutin; 4, Naringin; 5, Hesperidin; 6, Neohesperidin; 7, Rutin; 8, Rhoifolin; 9, Diosmin; 10, Didymin; 11, Hesperetin; 12, Luteolin; 13, Diosmetin; 14, Sinensetin; 15, Nobiletin; 16, Tangeretin) and samples. (**A**) Flavonoid standard mixture, 283 nm. (**B**) Flavonoid standard mixture, 330 nm. (**C**) PU, 283 nm. (**D**) CP, 330 nm. (**E**) PWS, 283 nm. (**F**) SE, 283 nm.

We use the HPLC method to analyze individual flavonoids in different components of CRC quantitatively and chose the 11 most abundant flavonoids (narirutin, hesperidin, neohesperidin, rutin, diosmin, didymin, hesperetin, diosmetin, sinensetin, nobiletin, tangeretin) in CRC for further study. HPLC chromatograms of the standards and samples are shown in Figure 7.

The determination of the limit of detection (LOD) and limit of quantification (LOQ) was conducted as part of the method validation process (Table 2). The LOD ranged from 0.06 to 46.24 μg/mL and the LOQ was between 0.18 and 140.14 μg/mL.

The content of flavonoids is summarized in Table 3. We observed that PU and PWS exhibited higher levels of hesperidin (7601.97 ± 140.13 μg/g DW and 7543.86 ± 24.93 μg/g DW, respectively), a flavonoid belonging to the flavanone group [44]. Further, hesperidin has been shown to reduce the severity of dextran sulfate sodium (DSS)-induced ulcerative colitis in mice, as evidenced by significant decreases in serum disease activity index (DAI), myeloperoxidase (MPO) activity, malondialdehyde (MDA) levels, and IL-6 [45]. The expression levels of Narirutin and Didymin (864.92 ± 8.33 μg/g DW and 359.66 ± 2.03 μg/g DW, respectively) were found to be higher in PU compared to other groups of samples. CP exhibited higher levels of polymethoxylated flavonoids such as Nobiletin and Tangeretin (3585.99 ± 26.70 μg/g DW and 2393.86 ± 20.61 μg/g DW, respectively), which corroborates the results of non-targeted metabolomics and confirms the accuracy and reliability of the metabolomics approach.

### 3.7. Overview of Total Phenolic Content and Antioxidant Activity in Different Parts of Citrus reticulata ‘Chachi’

The total phenol content we determined is shown in Table 3. The results showed that CP had the highest TPC (6.8 ± 0.13 mg GAE/g DW), whereas SE possessed the lowest TPC (2.16 ± 0.42 GAE/g DW).

Oxidative stress can be responsible for the induction of inflammation, leading to numerous chronic diseases [46]. Antioxidants are crucial substances that can help to reduce oxidative stress [18]. This study assessed the antioxidant potential of various *Citrus reticulata* ‘Chachi’ segments via DPPH, ABTS, and FRAP assays. The findings revealed robust antioxidant effects across all extracts, with notable disparities in activity levels (*p* < 0.05), as detailed in Table 3. The assays agreed in rating the antioxidant strength in CP, PU, PWS, and SE and the disparity in antioxidant potential is likely due to varying phenolic content [38]. The analysis showed that CP possessed the highest total phenolic content, corresponding with its antioxidant performance, while SE had the least. Interestingly, the literature also supports the strong antioxidant attributes of flavonoids [47]. Further, the metabolomic findings suggest that PU’s increased antioxidant activity compared to PWS may be attributed to its higher concentrations of hesperidin, isoeriocitrin, narirutin, and didymin.

### 3.8. Network Pharmacology Analysis

#### 3.8.1. Flavonoid Active Ingredients and Core Target Prediction

**Figure 8:**
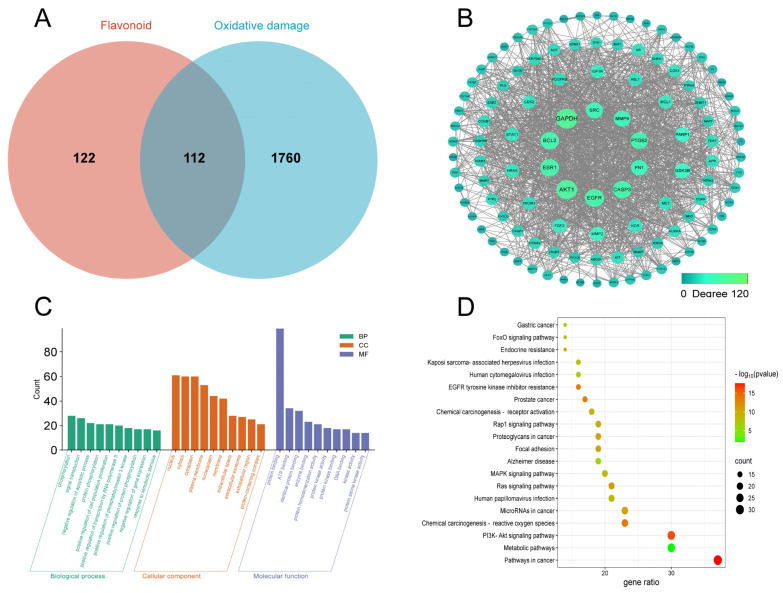
(**A**) Venn diagram of overlapping targets between flavonoid active components and oxidative damage. (**B**,**C**) Protein–protein interaction (PPI) analysis network diagram. (**C**) Top 10 GO enrichment analysis bar chart. (**D**) Top 20 KEGG enrichment analysis bubble chart of signaling pathways.

The 2D chemical structure of the 11 flavonoid active compounds detected by HPLC, as described above, were imported into the SwissTargetPrediction database to obtain the potential targets of each compound. A total of 122 relevant targets were obtained after removing duplicates (Figure 8A, Table S11). Further, we screened 1760 potential targets associated with oxidative damage from GeneCards and OMIM databases (Table S12). Then, a total of 112 overlapping targets of flavonoid active ingredients with oxidative damage were identified by plotting a Wayne diagram (Table S13). These 112 overlapping targets were input into the STRING database, and the resultant files were then imported into Cytoscape, which was used to draw a network diagram for PPI analysis. Further, we screened ten key targets, including GAPDH, EGFR, BCL2, PTGS2, ESR1, CASP3, MMP9, AKT1, FN1, and SRC (Figure 8B). These targets often play a crucial role in signaling pathways, potentially acting as significant action targets in the antioxidant response of CRC’s parts. Evidence suggests that GAPDH and its involvement in glycolysis mediate various functions that regulate oxidative stress [48,49]. Studies have found that phillygenin inhibits LPS-induced macrophage activation by downregulating GAPDH enzyme activity, thereby exerting anti-inflammatory effects [50]. Furthermore, pan-cancer species analysis has shown that GAPDH is commonly expressed in most cancer types and may contribute to various important cancer-related pathways and biological processes [51]. EGFR, a membrane-spanning glycoprotein, initiates multiple intracellular signaling pathways upon ligand binding, such as PI3K, MAPK, and ERK pathways [52,53]. The inhibition of EGFR or its ligands can induce tumor cell apoptosis by increasing ROS production and downregulating superoxide dismutase expression [54,55]. PTGS2, a COX isoform, is crucial for inflammatory responses via prostaglandin synthesis. MMP9, key in ECM degradation for cancer cell metastasis [56], can be targeted by flavonoids from *Citrus reticulata* ‘Chachi’ to inhibit tumor invasion and migration [57]. Flavonoids may reduce oxidative stress by modulating the expression of genes and proteins such as GAPDH, EGFR, BCL2, PTGS2, and ESR1.

#### 3.8.2. GO and KEGG Enrichment Analysis

GO and KEGG enrichment analyses were conducted on the overlapping targets to predict the functions and pathways of genes or proteins associated with the antioxidant effects of flavonoids in *Citrus reticulata* ‘Chachi’. The top ten GO terms for biological processes, cellular components, and molecular functions were identified, suggesting that these targets are mainly found in the nucleus, cytoplasm, and plasma membrane and are involved in functions such as protein binding, ATP binding, and enzyme binding, as well as processes such as protein phosphorylation, signal transduction, apoptosis inhibition, and cell proliferation promotion (Figure 8C). A bubble chart depicts the 20 most relevant pathways for oxidative damage, including cancer-related pathways, the PI3K-Akt pathway, ROS in chemical carcinogenesis, the Ras pathway, and the MAPK pathway (Figure 8D). The PI3K/Akt pathway, a crucial intracellular signaling cascade, regulates cell differentiation, metabolism, growth, and cytoskeletal rearrangement [58]. Oxidative stress elevates ROS levels, triggers the MAPK pathway, and can damage lipids, proteins, and DNA [59]. These findings suggest that flavonoids in *Citrus reticulata* ‘Chachi’ may mitigate oxidative damage by modulating the PI3K/Akt and MAPK pathways.

## 4. Conclusions

Our research findings indicate that *Citrus reticulata* ‘Chachi’ (CP, PU, PWS, SE) is rich in metabolites such as phenolics and terpenoids that promote human health and thereby possess potential economic value. By employing untargeted metabolomics combined with multivariate statistical methods for comparative analysis, we have identified significant metabolic differences among the four parts of the *Citrus reticulata* ‘Chachi’. A total of 1622 differentially expressed metabolites were identified, of which 816 secondary metabolites were selected, predominantly terpenoids, flavonoids, and phenolic acids. In the CP group, flavonoids and phenolic acids were abundant, particularly polymethoxyflavones (PMFs). The PU and PWS contained a higher content of flavonoids, mostly flavanones, which could serve as prominent bioactive components and have the potential for the development of functional foods. The SE group showed a richer content of terpenoids and steroids, establishing the seeds as a valuable source for extracting terpenoid substances. Further, KEGG enrichment analysis revealed that flavonoid biosynthesis is the primary metabolic pathway. The total phenolic content was found to be highest in CP, and the flavonoids such as nobiletin and tangeretin were most abundant, while hesperidin, narirutin, and didymin were most abundant in PU. Additionally, in vitro antioxidant experiments showed that extracts from all four parts of CRC exhibit strong antioxidant properties, and the activity was ranked as CP > PU > PWS > SE, suggesting that the total phenolic content might be the reason for the strength of the antioxidant activity. Finally, network pharmacology analysis predicted GAPDH, EGFR, BCL2, PTGS2, and ESR1 to be key targets for the antioxidant effects of flavonoids in CRC. GO and KEGG enrichment analyses revealed that flavonoids in CRC might exert their antioxidant effects by regulating pathways such as cancer-related pathways, the PI3K-Akt signaling pathway, chemical carcinogenesis, reactive oxygen species, the Ras signaling pathway, and the MAPK signaling pathway. Although in vitro studies have established the antioxidant activity of the components of CRC, further studies in animal models are needed to validate their in vivo bioactivity. Overall, these findings not only provide the scientific basis for guiding the future development of CRC processing products and the utilization of byproducts but also offer potential candidate components for the development of new health foods. 

## Figures and Tables

**Table 1:** Information of the Citrus Reticulata ‘Chachi’ samples used in the analysis.

Code	Citrus Part	Species	Source
CP	Citrus peel stored for 3 years	*Citrus Reticulata* ‘Chachi’	Jiangmen, Guangdong, China
PWS	Citrus pulp with seeds	*Citrus Reticulata* ‘Chachi’	Jiangmen, Guangdong, China
PU	Citrus pulp without seeds	*Citrus Reticulata* ‘Chachi’	Jiangmen, Guangdong, China
SE	Seeds	*Citrus Reticulata* ‘Chachi’	Jiangmen, Guangdong, China

**Table 2:** The limit of detection (LOD) and limit of quantification (LOQ) for flavonoids.

Flavonoids	λ (nm)	Retention Time (min)	LOD (μg/mL)	LOQ (μg/mL)
Narirutin	283	20.07	2.75	8.33
Hesperidin	283	22.50	46.24	140.14
Neohesperidin	283	24.09	0.06	0.18
Rutin	283	25.32	4.65	80.68
Diosmin	330	31.25	3.46	10.48
Didymin	283	32.98	0.67	2.03
Hesperetin	283	36.04	0.11	0.33
Diosmetin	330	39.87	0.19	0.56
Sinensetin	330	41.23	0.46	1.39
Nobiletin	330	43.43	0.46	1.40
Tangeretin	330	45.40	0.08	0.24

Values were expressed as 3.3 *σ/s* and 10 *σ/s* for LOD and LOQ, respectively. *σ* is the standard deviation of the regression line (μg/mL); *s* is the slope.

**Table 3:** Results of TPC, flavonoid compounds, ABTS, DPPH, and FRAP free radical scavenging activities for the four parts of CRC.

	PU	PWS	CP	SE
TPC (mg GAE/g DW)	4.52 ± 0.51 ^b^	4.26 ± 0.27 ^b^	6.8 ± 0.13 ^a^	2.16 ± 0.42 ^c^
Narirutin (μg/g DW)	864.92 ± 8.33 ^a^	746.06 ± 3.45 ^b^	290.27 ± 0.73 ^d^	410.04 ± 21.10 ^c^
Hesperidin (μg/g DW)	7601.97 ± 140.13 ^a^	7543.86 ± 24.93 ^a^	5754.18 ± 350.74 ^b^	1617.39 ± 20.6 ^c^
Neohesperidin (μg/g DW)	NA ^b^	NA ^b^	26.43 ± 0.18 ^a^	NA ^b^
Rutin (μg/g DW)	NA ^c^	115.15 ± 14.10 ^b^	NA ^c^	416.88 ± 18.01 ^a^
Diosmin (μg/g DW)	219.20 ± 10.48 ^b^	140.58 ± 0.32 ^c^	631.24 ± 10.80 ^a^	127.20 ± 20.59 ^c^
Didymin (μg/g DW)	359.66 ± 2.03 ^a^	287.54 ± 13.08 ^b^	105.17 ± 54.20 ^c^	157.76 ± 4.86 ^c^
Hesperetin (μg/g DW)	NA ^b^	NA ^b^	57.87 ± 11.21 ^a^	NA ^b^
Diosmetin (μg/g DW)	NA ^b^	NA ^b^	47.84 ± 0.25 ^a^	NA ^b^
Sinensetin (μg/g DW)	NA ^b^	NA ^b^	189.74 ± 1.40 ^a^	NA ^b^
Nobiletin (μg/g DW)	11.17 ± 0.56 ^c^	12.25 ± 0.28 ^c^	3585.99 ± 26.70 ^a^	43.33 ± 0.72 ^b^
Tangeretin (μg/g DW)	17.99 ± 1.39 ^c^	23.90 ± 0.30 ^c^	2393.86 ± 20.61 ^a^	47.65 ± 3.82 ^b^
ABTS (µmol Trolox/g DW)	1.00 ± 0.004 ^b^	0.97 ± 0.002 ^c^	1.02 ± 0.001 ^a^	0.91 ± 0.002 ^d^
DPPH (µmol Trolox/g DW)	0.5 ± 0.004 ^b^	0.47 ± 0.001 ^c^	0.56 ± 0.002 ^a^	0.23 ± 0.006 ^d^
FRAP (µmol Trolox/g DW)	0.64 ± 0.004 ^b^	0.53 ± 0.003 ^c^	1.12 ± 0.003 ^a^	0.42 ± 0.005 ^d^

Different lowercase letters indicate significant differences between groups (*p* < 0.05); NA—not detected.

## Data Availability

The original contributions presented in this study are included in the article/Supplementary Materials. Further inquiries can be directed to the corresponding authors.

## Supplementary Materials

The following supporting information can be downloaded at https://www.mdpi.com/article/10.3390/foods13244018/s1, Figure S1. Identification of Metabolites; Figure S2. Q2 and R2Y values; Table S1. The overlapping targets were submitted to the David database; Table S2. Differential metabolism statistics; Table S3. The explanation of data variability by the two principal components; Table S4. The levels of Flavonoids in CP VS PU; Table S5. The levels of Flavonoids in PWS VS PU; Table S6. The levels of Flavonoids in SE VS PU; Table S7. The levels of Phenolic acids and derivatives in CP VS PU; Table S8. The levels of Phenolic acids and derivatives in PWS VS PU; Table S9. The levels of Phenolic acids and derivatives in SE VS PU; Table S10. The levels of Alkaloids in CP VS PU; Table S11. Overlapping targets between flavonoid active components and oxidative damage; Table S12. 1760 potential targets associated with oxidative damage from GeneCards and OMIM databases; Table S13. A total of 112 overlapping targets of flavonoid active ingredients with oxidative damage.
