# Supplementary figures and images for: Antioxidant Effects and Potential Mechanisms of Citrus reticulata ‘Chachi’ Components: An Integrated Approach of Network Pharmacology and Metabolomics
Source: Foods. 2024 Dec 12;13(24):4018. doi: 10.3390/foods13244018 (PMC11675786; doi:10.3390/foods13244018)

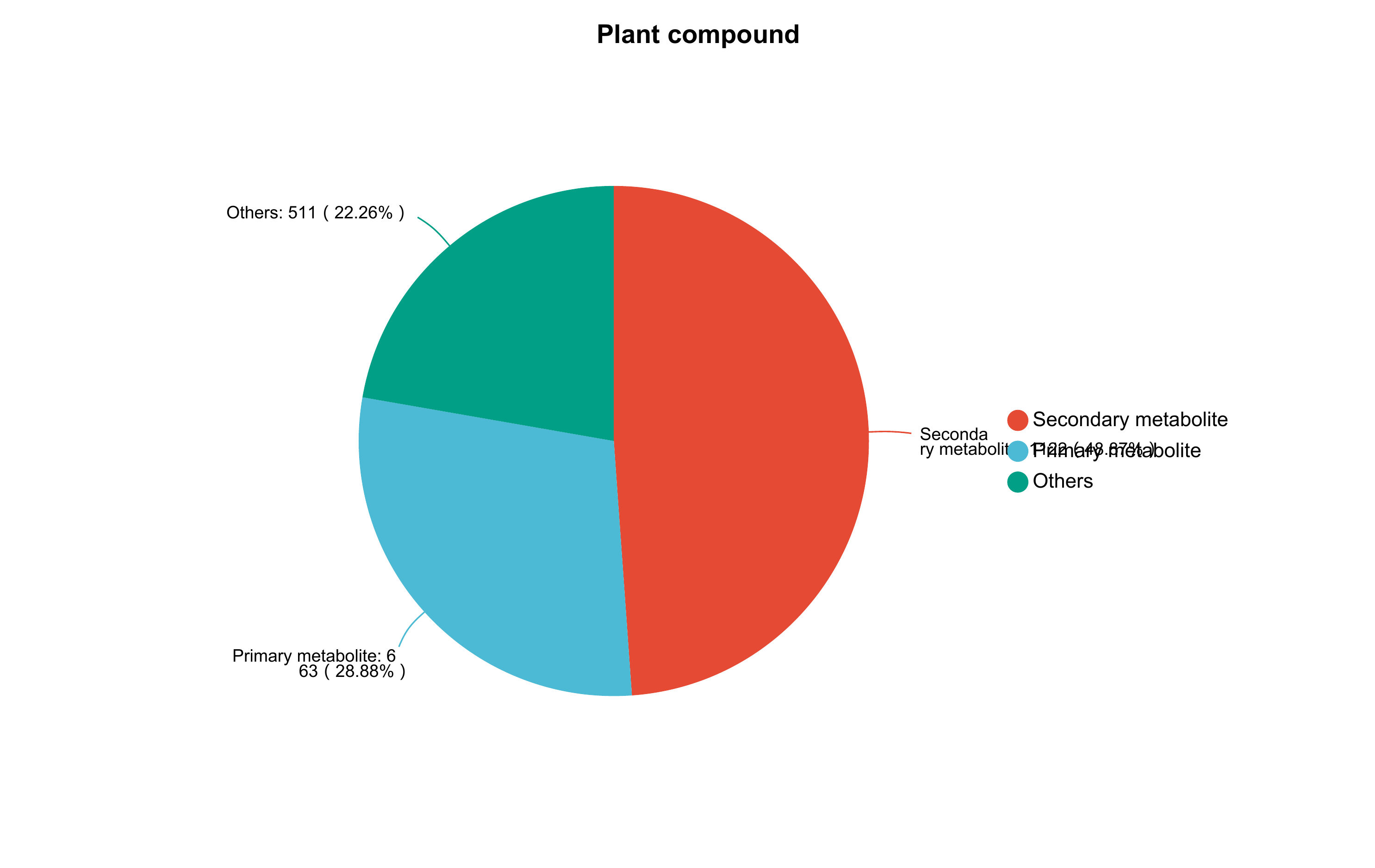

Supplement: Supplementary file 1 [file foods-13-04018-s001.zip › Supplementary materials/Figure S1.Identification of Metabolites.tiff]

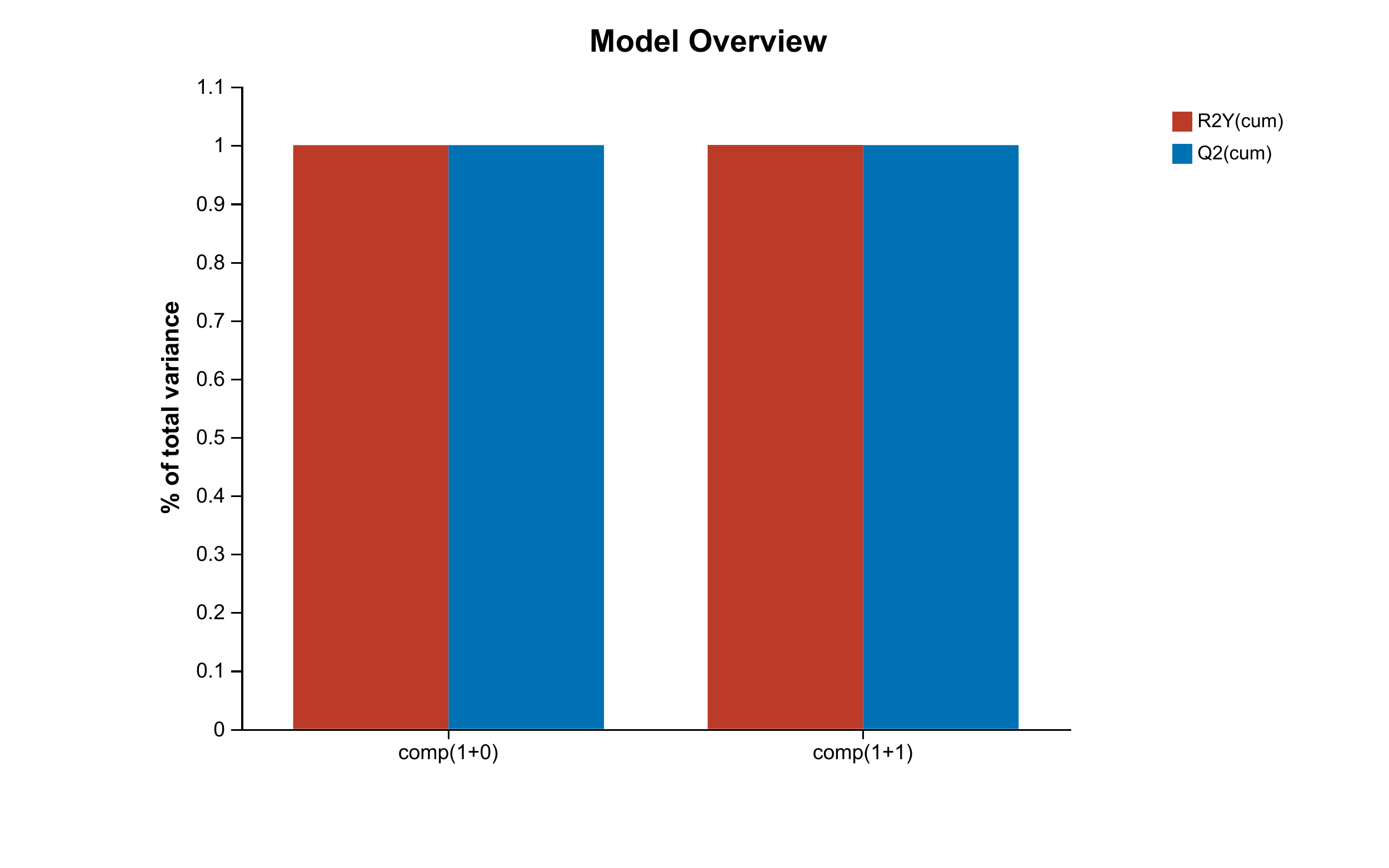

Supplement: Supplementary file 1 [file foods-13-04018-s001.zip › Supplementary materials/Figure S2.Q2 and R2Y values.tiff]
